# CRISPR/Cas9-Mediated Resistance to Wheat Dwarf Virus in Hexaploid Wheat (*Triticum aestivum* L.)

**DOI:** 10.3390/v16091382

**Published:** 2024-08-29

**Authors:** Xiaoyu Yuan, Keya Xu, Fang Yan, Zhiyuan Liu, Carl Spetz, Huanbin Zhou, Xiaojie Wang, Huaibing Jin, Xifeng Wang, Yan Liu

**Affiliations:** 1 State Key Laboratory for Biology of Plant Diseases and Insect Pests, Institute of Plant Protection, Chinese Academy of Agricultural Sciences, Beijing 100193, China; yxy202103@163.com (X.Y.); xukkkkkya@163.com (K.X.); yanfang15108@163.com (F.Y.); lzy1561176762@163.com (Z.L.); zhouhuanbin@caas.cn (H.Z.); jinhuaibing@caas.cn (H.J.); 2 Norwegian Institute of Bioeconomy Research, Hoegskoleveien 7, 1432 Ås, Norway; carl.spetz@nibio.no; 3 State Key Laboratory of Crop Stress Biology for Arid Areas, College of Plant Protection, Northwest A&F University, Yangling 712100, China; wangxiaojie@nwsuaf.edu.cn

**Keywords:** wheat dwarf virus, *Triticum aestivum*, CRISPR/Cas9, resistance

## Abstract

Wheat dwarf virus (WDV, genus *Mastrevirus*, family *Geminiviridae*) is one of the causal agents of wheat viral disease, which severely impacts wheat production in most wheat-growing regions in the world. Currently, there is little information about natural resistance against WDV in common wheat germplasms. CRISPR/Cas9 technology is being utilized to manufacture transgenic plants resistant to different diseases. In the present study, we used the CRISPR/Cas9 system targeting overlapping regions of coat protein (CP) and movement protein (MP) (referred to as CP/MP) or large intergenic region (LIR) in the wheat variety ‘Fielder’ to develop resistance against WDV. WDV-inoculated T_1_ progenies expressing Cas9 and sgRNA for CP/MP and LIR showed complete resistance against WDV and no accumulation of viral DNA compared with control plants. Mutation analysis revealed that the CP/MP and LIR targeting sites have small indels in the corresponding Cas9-positive plants. Additionally, virus inhibition and indel mutations occurred in T_2_ homozygous lines. Together, our work gives efficient results of the engineering of CRISPR/Cas9-mediated WDV resistance in common wheat plants, and the specific sgRNAs identified in this study can be extended to utilize the CRISPR/Cas9 system to confer resistance to WDV in other cereal crops such as barley, oats, and rye.

## 1. Introduction

Wheat (*Triticum aestivum* L.) is the most important cereal food crop in the world; however, it is susceptible to various pathogens. More than 50 viruses infecting wheat have been reported worldwide [1]. Wheat dwarf virus (WDV), a geminivirus transmitted by the leafhopper *Psammotettix alienus*, has caused severe damage to cereal crops, including wheat, barley, and oats, worldwide [2,3,4]. Its genome consists of a monopartite, single-stranded (ss), circular DNA separated by a large intergenic region (LIR) and a small intergenic region (SIR), encoding the coat protein (CP) and the movement protein (MP) on the virion strand and the two replication-related proteins RepA and Rep on the complementary strand [5,6]. There is currently little information on natural resistance to WDV in wheat germplasm [7]. In recent years, CRISPR/Cas-mediated genome editing has emerged as a powerful technology of choice for next-generation plant breeding to confer resistance to DNA or RNA viruses by targeting either the viral genome or susceptibility factors of the host plant genome [8]. Most studies on the use of the CRISPR/Cas9 system to engineer resistance to geminiviruses have been conducted in model plants and crops [9,10,11,12,13,14]. Upon geminivirus infection, viral DNA replicates by forming replication intermediates of double-stranded DNA (dsDNA) within the nucleus of the infected plant cell [15]. The Cas9-sgRNA complexes target viral dsDNA and cleave DNA target sites, thereby inhibiting viral replication and conferring resistance to DNA virus infection in plants [12]. In barley, CRISPR/Cas9 systems containing combinations of multiplexed guide RNAs (gRNAs) have been successfully applied to target conserved regions of the WDV genome and confer effective resistance to WDV [16]; however, to date, the application of the CRISPR/Cas9 system in common wheat against WDV has not been reported. In this study, we investigated the ability of the CRISPR/Cas9 system delivered by Agrobacterium to target WDV in common wheat and obtained transgenic wheat germplasm resistant to WDV.

## 2. Materials and Methods

### 2.1. Virus Propagation and Leafhopper Inoculation

The WDV isolate SXHC10-17 (accession number JQ647499) used in this experiment was originally collected from an infected wheat plant in Hancheng, Shaanxi Province, in 2012, and maintained on susceptible wheat plants (cultivars Yangmai 12). The vector leafhopper *Psammotettix alienus* used in this experiment, the progeny of a population collected from Langfang, Hebei Province, in April 2016, was continuously reared on wheat seedlings in an insect-proof cage at 23 ± 1 °C under a 16:8 h light/dark photoperiod and 40–60% relative humidity and transferred to fresh seedlings every 4 weeks. Third-instar leafhoppers were allowed to feed on WDV-infected wheat plants for at least a 96 h acquisition access period (AAP), and then WDV-viruliferous leafhoppers were applied to selected transgenic and control seedlings at the two-leaf stage to perform a 96 h inoculation procedure. All plants were further grown in a climate chamber at 20–22 °C.

### 2.2. sgRNA Design and Construction of CRISPR/Cas9 Expression Vector

A 20 bp sgRNA sequence targeting the CP or LIR conserved genome sequences of WDV isolate SXHC10-17 and other isolates was designed using the web tool Benchling (https://www.benchling.com/crispr) (accessed on 6 August 2021), driven by the wheat U6 gene promoter. Off-target possibilities were excluded using a blast search against the wheat reference genome (IWGSC RefSeq v2.1) in the Ensembl Plants database. The Cas9 gene used in this plasmid with a bialaphos resistance (*BAR*) screening gene was wheat-codon-optimized and driven by the maize ubiquitin promoter [17].

### 2.3. Agrobacterium-Mediated Wheat Transformation

Using Agrobacterium-mediated transformation, we introduced the experimental constructs into wheat cultivars ‘Fielder’ according to a previously published protocol [18], with slight modifications. Briefly, the CRISPR/Cas9 expression vector was transformed into *Agrobacterium tumefaciens* strain EHA105 via electroporation. The immature embryos were isolated and incubated with *Agrobacterium tumefaciens* strain EHA105 harboring the expression vector for 5 min. After cocultivation at 25 °C for 2 d in the dark, the immature embryos were transferred to plates. The explants were then cultured for callus induction under selective conditions using *Bialophos*; this was followed by plant regeneration. The rooted plantlets were then transferred to pots and grown in growth chambers at 20 °C with light intensity greater than 60,000 lx and 16 °C under darkness. 

### 2.4. Transcriptional Analysis Using sqRT-PCR and RT-qPCR

Total RNA was isolated from wheat leaves at different time points using TRIzol reagent (Invitrogen, Life Technologies, Carlsbad, CA, USA) according to the manufacturer’s instructions. To quantify the expression of the viral Rep gene in transgenic plants, mRNA levels of the Rep gene were initially explored using semi-quantitative reverse transcription and polymerase chain reaction (sqRT-PCR) [19]. In addition, first-strand cDNA was synthesized using EasyScript^®^ All-in-One First-Strand cDNA Synthesis SuperMix for qPCR (TransGen, Beijing, China), and RT-qPCR was performed using Hieff UNICON^®^ Universal Blue qPCR SYBR Green Master Mix (Yeasen, Shanghai, China) on an Applied Biosystems 7500 Real-Time PCR System (Thermo Fisher Scientific, Waltham, MA, USA). Each sample was analyzed in triplicate. Specific primers were designed to detect the abundance of the viral Rep gene and the expression of sgRNA. Wheat glyceraldehyde-3-phosphate dehydrogenase (GAPDH) was used as the control gene [20]. Primer information is provided in Supplementary Table S1.

### 2.5. Western Blot Assay

Cas9 expression was determined by Western blotting. Total protein was extracted from two independent lines transformed with sgLIR and sgCP/MP. Protein from each sample (10 μg per lane) was loaded onto a 4–20% SDS-PAGE gel, electrophoretically separated, and transferred to a polyvinyl difluoride (PVDF) transfer membrane. Anti-FLAG antibody (ABclonal, Wuhan, China) was used as a primary antibody (1:1000 dilution) to detect FLAG-tagged TaCas9. Horseradish peroxidase (HRP) anti-mouse secondary antibody (TransGen, Beijing, China) was used at a 1:2500 dilution. Rubisco large subunit protein (RbcL) stained with Ponceau S staining solution was used as a loading control. The blotted membranes were washed thoroughly and visualized by chemiluminescence using a commercial ECL detection system (KPL, Gaithersburg, MD, USA).

### 2.6. Northern Blot Analysis

Northern blot analysis was performed according to the protocol reported by Liu et al. (2014) [6]. Briefly, 20 μg total RNA for each sample was separated by electrophoresis for 14 h in 1.2% formaldehyde–agarose gels and transferred to a nylon membrane (Cytiva, Marlborough, MA, USA). A 453 bp WDV DNA probe was synthesized and labeled using the PCR DIG kit (Roche, Basel, Switzerland). RNA accumulation was detected using a Touch Imager (e-Blot, Shanghai, China).

### 2.7. Detection of Edited Mutations

To identify the predominant types of insertions and deletions (indels) in the region flanking the gRNA target site, WDV genome from all virus-inoculated leaves was amplified using two sets of primers, WDV-554-F/WDV-554-R and WDV-773-F/WDV-773-R, using 2×Hieff^®^ Ultra-Rapid HotStart PCR Master Mix (Yeasen, Shanghai, China). Amplicons for each sample were sequenced and the sequences were analyzed using Hi-TOM software (version 1.0) [21]. To confirm the presence of the targeted mutation, the PCR products of the sgRNA for each target were subcloned into the TA/Blunt-Zero cloning vector (Vazyme, Nanjing, China). A total of 7–10 colonies from each line were randomly selected and subjected to Sanger sequencing (Sangon Biotech Co., Ltd., Shanghai, China). Mutations were identified by comparing them with the wild-type (WT) sequences of corresponding sgRNAs. The primers used in this study are listed in Table S1. 

## 3. Results

**Figure 1:**
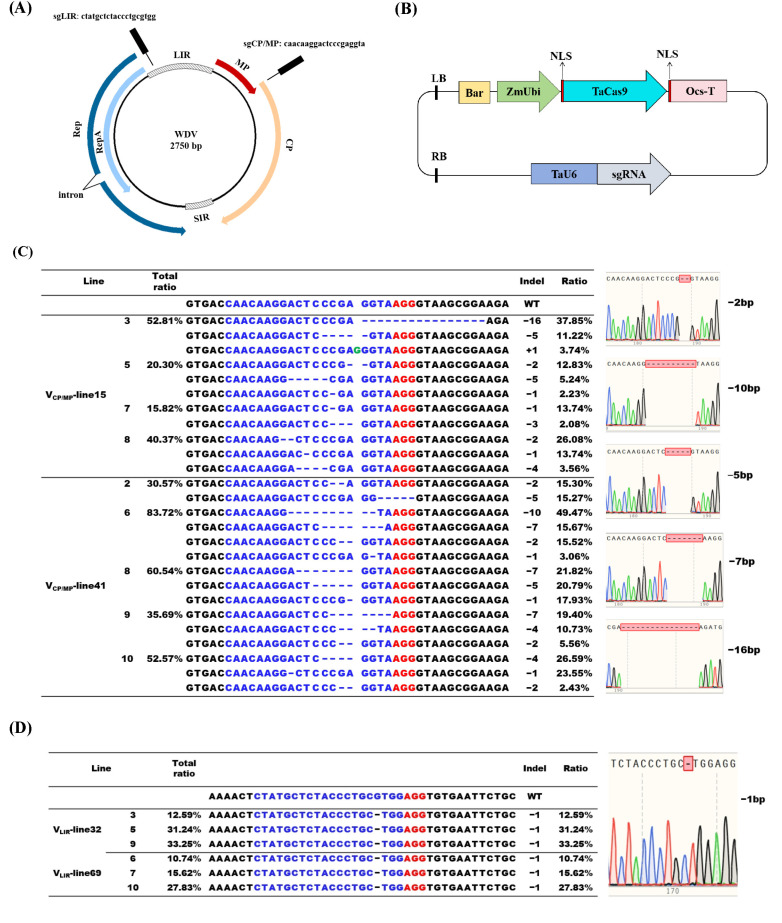
CRISPR/Cas9-mediated wheat dwarf virus (WDV) interference in hexaploid wheat. (**A**) WDV genome structure and positions of the sgRNA target sites; (**B**) schematic diagram of the CRISPR/Cas9 system constructed in this study; (**C**,**D**) Hi-TOM sequence analysis of CP/MP-sgRNA and LIR-sgRNA-Cas9-induced mutations at the target sites and the representative Sanger sequencing chromatographs at 7 dpi. WT, Wild-type control. PAM is in red and gRNAs in blue. Green letters indicate inserted nucleotides. Blue dashes denote nucleotide deletions. −/+ indicates deletion/insertion of nucleotides.

First, two sgRNAs containing a 5′-NGG-3′ protospace adjacent motif (PAM) were designed, one targeting the promoter DNA of the LIR (designated sgLIR) and the other targeting the CP/MP overlap region (designated sgCP/MP) within the conserved sequence of WDV isolates (Figure 1A). Then, spacer sequences with relatively high GC contents and no detectable off-targets were selected. Next, two oligonucleotides complementary to the sgRNA spacer sequence were synthesized, annealed to form a double-stranded DNA fragment, and then inserted into the *Bsa*I sites of a Gateway entry vector pTagRNA4 via restriction enzyme digestion to generate CP/MP or LIR sgRNA clones. After *Apa*I digestion, the sgRNA expression cassettes TaU6-sgCP/MP and TaU6-sgLIR were shuttled into pUbi-TaCas9 via LR Gateway recombination to generate plasmids pUbi-TaCas9-sgCP/MP and pUbi-TaCas9-sgLIR, respectively (Figure 1B). All primers used for vector construction are listed in Table S1. 

The construct containing either sgCP/MP- or sgLIR-Cas9 was individually transformed into the wheat cultivar Fielder using Agrobacterium-mediated transformation methods. A total of 51 and 89 T_0_ plants were, respectively, regenerated from the transformed calli of the constructs sgCP/MP or sgLIR after transgenic plants were identified by their resistance to the *BAR* gene. Further, PCR screening using Cas9-specific primers showed that 78.4% and 43.8% of sgCP/MP- and sgLIR-Cas9 transgenic plants, respectively, were positive. These results indicate that sgCP/MP- and sgLIR-Cas9 constructs successfully achieved high wheat transformation efficiencies.

We selected two lines (C15 and C41) of sgCP/MP transformants and two lines (L32 and L69) of sgLIR transformants and challenged their T_1_ progeny plants with WDV via inoculation with viruliferous leafhoppers. Wild-type (WT) plants were used as controls. Seven days later, both WDV and Cas9-positivity were confirmed via PCR of test plants using specific primers (primer information detailed in Table S1). Further, Hi-TOM sequencing of the PCR amplicons using primers flanking each target site was used to survey the mutations in the DNA of samples C15, C41, L32, and L69. The INDEL percentage was calculated based on the sequence reads. Indels at the predicted cleavage sites of sgCP/MP or sgLIR in the different lines were present at frequencies of >15% and 27%, respectively (Figure 1C). The majority of sgCP/MP-induced mutations were 10 or 16 bp deletions. In contrast to indels at the CP/MP region, the LIR of the WDV had only 1 bp deletion (no long deletions) at the targeted sites produced by non-homologous end joining (NHEJ). Sanger sequencing further confirmed the presence of these site-specific indels in both the CP/MP region and LIR of the WDV genome (Figure 1D). These results clearly indicate that gene editing of the WDV genome occurs in all four transgenic lines of T_1_ plants.

**Figure 2:**
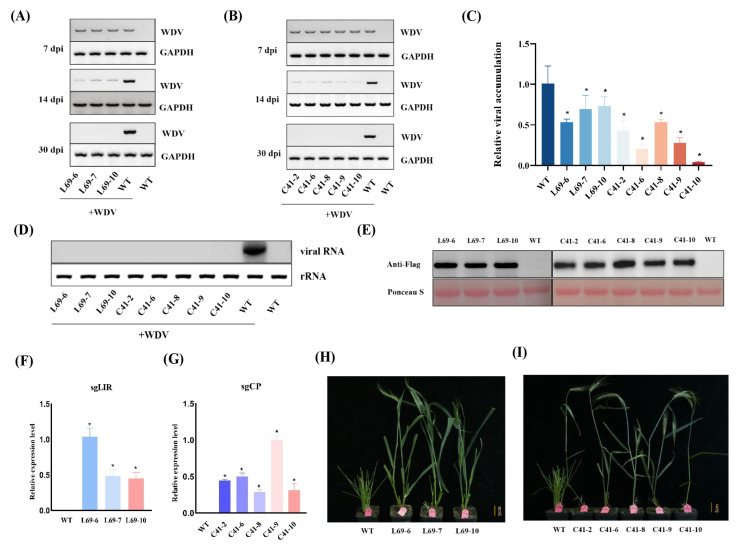
Molecular and phenotypic evidence of WDV resistance in T_1_ generation of lines L69 and C41. (**A**,**B**) mRNA expression levels of the viral Rep gene in transgenic lines L69 and C41 were examined using semi-quantitative reverse transcription PCR (sqRT-PCR) assay at the indicated time points; (**C**) WDV accumulation in systemic leaves at 14 dpi were assessed using reverse transcription-quantitative PCR (RT-qPCR). Error bars represent SD, asterisks indicate significance, * *p* < 0.05. Data are representative of three biological replicates; (**D**) Northern blot confirms the absence of WDV in systemic leaves at 30 dpi. Ethidium bromide staining of ribosomal RNAs (rRNA) was used as loading controls; (**E**) Western blot confirmation of the Cas9 protein at 30 dpi using an anti-FLAG primary antibody. Ponceau-S-stained Rubisco served as a loading control; (**F**,**G**) Expression level of sgRNAs in wheat systemic leaves at 30 dpi was assessed using RT-qPCR; (**H**,**I**) symptoms of WDV in the transgenic lines L69 and C41 in comparison with wild-type wheat plants at 80 dpi.

Next, mutant plants from line C41 and line L69 were selected for further molecular and biological assays. As shown in Figure 2A,B, mRNA transcription levels of WDV in these eight transgenic plants (C41-2, -6, -8, -9, and -10 and L69-6, -7, and -10) were similar to those of inoculated WT plants at 7 dpi based on sqRT-PCR analysis. In contrast, virus accumulation in systemic leaves of C41 and L69 transgenic plants at 14 dpi was significantly reduced compared with inoculated WT plants. Quantitative reverse transcription PCR (RT-qPCR) also showed that both the sgCP/MP and sgLIR constructs inhibited WDV replication to varying levels (25–55% in C41 plants and 48–97% in L69 plants) (Figure 2C). At 30 dpi, dwarfism symptoms were observed in the infected control plants. On the contrary, the transgenic plants (C41-2, -6, -8, -9, and -10, and L69-6, -7, and -10) did not show any visible symptoms (Figure S1). Consistent with the symptoms, sqRT-PCR analysis (Figure 2A,B) and Northern blotting (Figure 2D) further confirmed that there was no accumulation of WDV in the transgenic plants. High-level expression of Cas9 protein was demonstrated in T_1_ plants of both CP/MP- and LIR transgenic plants based on Western blot analysis using the anti-FLAG tag antibody (Figure 2E). Analysis of sgRNA expression levels using RT-qPCR showed that sgRNA was adequately expressed in systemic leaves of CP/MP and LIR transgenic plants (Figure 2F,G). At 80 dpi, strong disease symptoms such as severe dwarfism, yellowing, reduced available tillering, and sterility were observed in the control plants. By contrast, T_1_ transgenic plants (C41-2, -6, -8, -9, and -10, and L69-6, -7, and -10) stayed green and grew normally, indicating that high virus resistance was achieved in these plants (Figure 2H,I). These results demonstrate that both CP/MP- and LIR-Cas9 transgenic plants can reduce WDV accumulation compared to wild-type plants.

Further, we examined symptoms and accumulation of WDV in T_2_ homozygous lines of L69-10 and C41-6. As expected, all the T_2_ transgenic plants exhibited stable resistance to WDV. Similar to the results for T_1_ transgenic plants, more than four mutation types (−1, −2, −3, and −5 bp deletions) were detected in sgCP/MP in the T_2_ generation, while only 1 bp deletion was detected in sgLIR in the T_2_ generation (Figure S2). The transgenic-positive lines were indistinguishable from the wild-type wheat plants, indicating that the presence of the transgene cassette does not interfere with normal development. Taken together, these experiments demonstrate that T_2_ plants retain Cas9 activity and cleave viral DNA under the guidance of sgCP/MP or sgLIR.

## 4. Discussion

Previous studies have shown that overexpression of sgRNA-Cas9 specifically targeting non-coding and coding protein sequences in the virus genome is an effective way of generating new germplasm with high resistance to plant viruses. Here, we verify the application of CRISPR/Cas9 for engineering WDV resistance in common wheat. The sgRNA-Cas9 constructs targeting the CP/MP region or LIR achieved a reduction in viral load, and no WDV symptoms were subsequently observed in the corresponding transgenic plants.

Currently, next-generation sequencing (NGS) is increasingly being used for mutation screening [22,23,24], expected for T7 endonuclease I (T7EI) cleavage assays, and PCR-RE analysis and Sanger sequencing. In our work, the Hi-TOM platform was chosen for high-throughput analysis to track CRISPR/Cas9-mediated mutations in the WDV genome in the inoculated leaves of transgenic plants at 7 dpi. As shown in the Section 3, chimeric mutations in CP/MP target sites were accurately identified using the Hi-TOM platform. Based on 1.5 Gb of sequencing data, the overall efficiency of sgRNA targeting the CP/MP region was calculated to be up to 83.72%. It was also found that sgRNA targeting the LIR had only one mutation type and the highest efficiency of 33.25%. Our study showed that the type or frequency of mutations in the target sites of CP/MP and LIR were quite different. These results suggest that Hi-TOM sequencing is a promising approach for the high-throughput identification of viral mutations induced by CRISPR/Cas systems.

In 2019, Kis et al. [16] demonstrated that a single CRISPR/Cas9 construct containing multiplexed sgRNAs could confer effective resistance to WDV in barley; however, among the selected sgRNAs targeting CP/MP, Rep/RepA, and LIR in the WDV genome, only sgRNA targeting the Rep/RepA region mediated effective resistance. In the present study, specific sgRNAs targeting CP/MP and LIR sequences were selected and successfully shown to be effective. Both sgCP/MP and sgLIR were able to mediate targeted cleavage of the WDV genome and induce mutagenesis using the CRISPR/Cas9 system. Protein prediction analysis revealed that NHEJ-induced mutations generated at CP/MP targets can lead to premature termination of protein translation, thereby affecting protein function. The LIR of all geminiviruses is known to contain the origin of replication and promoter sequences for RNA polymerase II. The CRISPR/Cas9 system specifically targeting the IR has been successfully applied to inhibit tomato yellow leaf curl virus (TYLCV) infection in *Nicotiana benthamiana* [12]. It has been speculated that the inhibition of viral RNA transcripts is due to Cas9 binding rather than cleavage. We agree with this view that Cas9 occupancy or modification of IR renders it inaccessible to Rep and/or other binding proteins responsible for viral replication inhibition. 

Many studies, including this one, have shown that the geminivirus genome can be repaired at the target site by NHEJ. However, NHEJ is error-prone and leads to random nucleotide substitutions or indels, which may result in the production of newly evolved viral variants. The use of multi-targeting CRISPR sgRNAs is an efficient way of eliminating mutant viruses (escape mutants) that arise from CRISPR/Cas9-mediated genome editing. In Conclusion, we believe that the CRISPR/Cas9 system has great potential to confer stable resistance to WDV disease in cereal plants by directly targeting non-coding regions or multiple sites in the viral genome.

## Data Availability

All data are contained in the manuscript, figures, and Supplementary Figures.

## Supplementary Materials

The following supporting information can be downloaded at: https://www.mdpi.com/article/10.3390/v16091382/s1, Table S1: PCR primers used and their applications; Figure S1: Symptoms in the transgenic T_2_ lines and control plants at 30 dpi; Figure S2: Hi-TOM sequencing analysis of sgRNA-Cas9-induced virus mutations in T_2_ progenies of lines C41-6 and L69-10.
