# Supplementary material for: CRISPR/Cas9-Mediated Resistance to Wheat Dwarf Virus in Hexaploid Wheat (Triticum aestivum L.)
Source: Viruses. 2024 Aug 29;16(9):1382. doi: 10.3390/v16091382 (PMC11436044; doi:10.3390/v16091382)
Supplement: Supplementary file 1 [file viruses-16-01382-s001.zip › viruses-3156360-supplementary.pdf]

# Supplementary material

## CRISPR/Cas9-Mediated Resistance to Wheat Dwarf Virus in Hexaploid Wheat (*Triticum aestivum* L.)

Xiaoyu Yuan[1,†], Keya Xu[1, †] , Fang Yan[1], Zhiyuan Liu[1], Carl Spetz[2], Huanbin Zhou[1], Xiaojie Wang[3], Huaibing Jin[1], Xifeng Wang[1,*], and Yan Liu[1,*]

[1] State Key Laboratory for Biology of Plant Diseases and Insect Pests, Institute of Plant Protection, Chinese Academy of Agricultural Sciences, Beijing 100193, China;

[2] Norwegian Institute of Bioeconomy Research, Hoegskoleveien 7, 1432 ÅS, Norway;

[3] State Key Laboratory of Crop Stress Biology for Arid Areas, College of Plant Protection, Northwest A&F University, Yangling 712100, China

* Correspondence: wangxifeng@caas.cn (X.W.); liuyan06@caas.cn (Y.L.)

[†] These authors contributed equally to this work.

## Table S1 PCR Primers used and their applications

| Primer name | Primer sequence（5'-3'） | Application |
|---|---|---|
| gWDV-LIR-F1<br>gWDV-LIR-R1 | actcgTATGCTCTACCCTGCGTGG<br>aaacCCACGCAGGGTAGAGCATAc | To clone WDV LIR-sgRNA |
| gWDV-CP-F1<br>gWDV-CP-R1 | actcgAACAAGGACTCCCGAGGTA<br>aaacTACCTCGGGAGTCCTTGTTc | To clone WDV CP/MP-sgRNA |
| TaCas9-F1<br>TaCas9-R1 | GGTCTGGAGGATGCCCTTCT<br>GGCTATCGTGGACCTCCTGT | PCR detection for Cas9 |
| WDV-Rep-F<br>WDV-Rep-R | TGGTCTCATGGCAAATGAGTG<br>TCCATCACCAATCCCAATGCC | PCR detection for Rep of WDV |
| WDV-Rep-Q4-F<br>WDV-Rep-Q4-R | TGAGTGGGGAACATTCGTGG<br>GAAATAGATGGCGTGCCGTG | RT-qPCR for WDV accumulation |
| LIR-Q1-F<br>LIR-Q1-R | AAAACCCACGCAGGGTAGAG<br>CATCGGCTAGCGAAAGGGAA | RT-qPCR for sgRNA-LIR expression |
| CP-Q2-F<br>CP-Q2-R | GCGCGTGGCTTATAAACTGG<br>ACTATTTTGGGCCACACCGA | RT-qPCR for sgRNA-CP/MP expression |
| GAPDH-Q-F<br>GAPDH-Q-R | ACTTCCAGGGTGACAACAGG<br>GTGCTGTATCCCCACTCGTT | RT-qPCR for GAPDH expression |
| WDV-CP-Det-F<br>WDV-CP-Det-R | TGTCCGATGCATCAGCTCCGT<br>TAACACGCGTGCGTATAGGCA | Amplifying WDV probe for northern blot |
| CP-Hi-F2<br><br>CP-Hi-R2 | GGAGTGAGTACGGTGTGCAGGACCGAGG<br>AAATTGGTTACG<br>GAGTTGGATGCTGGATGGCGGCCTTACCC<br>TGAGCAAAA | Hi-TOM sequencing for CP/MP target site |
| LIR-Hi-F1<br><br>LIR-Hi-R1 | GGAGTGAGTACGGTGTGCAACAGCAGCG<br>ATGTAGAGG<br>GAGTTGGATGCTGGATGGGGACACATTGC<br>ATTTGCAGTG | Hi-TOM sequencing for LIR target site |
| WDV-554-F<br>WDV-554-R | TGTGTTTGCATCGGAGTCAACC<br>GCCCACACGATTCGCTACG | Mutation analysis in LIR target site |
| WDV-773-F<br>WDV-773-R | GTAGGCGTTGCTTGGCTTGC<br>TAATGTCGCCTATCTTGCCGTC | Mutation analysis in CP/MP target site |

(A) (B)

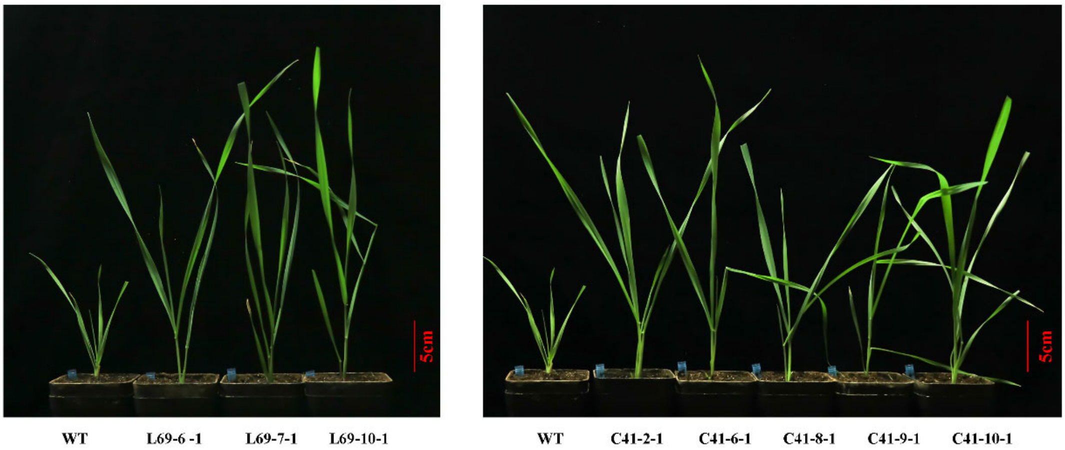


Figure S1. Symptoms in the transgenic $T_2$ lines and control plants at 30 dpi. WT, wild-type plant.

| Line | | Total ratio | | Indels | Ratio |
|---|---|---|---|---|---|
| $V_{CP}$-line41-6 | | | CAGATTAAATGGTGACCAACAAGGACTCCCGAGGTAAGGGTAAGCGGAAGATGGAAG | WT | |
| | 1 | 48.34% | CAGATTAAATGGTGACCAACAAGGACTC-----GTAAGGGTAAGCGGAAGATGGAAG | −5 | 41.08% |
| | | | CAGATTAAATGGTGACCAACAAGGAC-----AGGTAAGGGTAAGCGGAAGATGGAAG | −5 | 7.26% |
| | 6 | 16.97% | CAGATTAAATGGTGACCAACAAGGACTCCCG--GTAAGGGTAAGCGGAAGATGGAAG | −2 | 9.91% |
| | | | CAGATTAAATGGTGACCAACAAGGACTCCCG-GGTAAGGGTAAGCGGAAGATGGAAG | −1 | 2.75% |
| | | | CAGATTAAATGGTGACCAACAAGGACTCCCG-GGTAAGGGTAAGCGGAAGATGGAAG | −1 | 2.23% |
| | | | CAGATTAAATGGTGACCAACAAGGACTCCCG-GGTAAGGGTAAGCGGAAGATGGAAG | −3 | 2.08% |
| $V_{LIR}$-line69-10 | | | GGTGTGCCAGAAAACTCTATGCTCTACCCTGCGTGGAGGTGTGAATTCTGCACACTG | WT | |
| | 3 | 28.62% | GGTGTGCCAGAAAACTCTATGCTCTACCCTGC-TGGAGGTGTGAATTCTGCACACTG | −1 | 28.62% |
| | 5 | 39.33% | GGTGTGCCAGAAAACTCTATGCTCTACCCTGC-TGGAGGTGTGAATTCTGCACACTG | −1 | 39.33% |

**Figure S2.** Hi-TOM sequencing analysis of sgRNA-Cas9-induced mutations in T2 progenies of lines C41-6 and L69-10. WT, Wild-type control, PAM is in red and gRNAs in purple. Black dashes denote nucleotide deletions. −/+ indicates deletion/insertion of nucleotides.
